# Maggot Therapy in Wound Healing: A Systematic Review

**DOI:** 10.3390/ijerph17176103

**Published:** 2020-08-21

**Authors:** Mohd Zurairie Mohd Zubir, Samantha Holloway, Norhayati Mohd Noor

**Affiliations:** 1 School of Medicine, Cardiff University, Cardiff CF14 4YS, UK; zurairiezubir@gmail.com (M.Z.M.Z.); HollowaySL1@cardiff.ac.uk (S.H.); 2 Department of Family Medicine, School of Medical Sciences, Universiti Sains Malaysia, Kubang Kerian 16150, Kelantan, Malaysia

**Keywords:** maggots, wounds, hydrogel dressings, disinfection, granulation tissue, pain

## Abstract

Background: It is estimated that 2% of the population in developing countries suffer from a chronic wound, making it a hidden phenomenon that is increasing as populations age. The ease of access to maggot therapy has made it increasingly attractive for implementation. This study aimed to explore the effectiveness of maggot therapy as compared to hydrogel dressings in the healing of chronic wounds. Methods: An electronic literature search until October 2019 was performed using Medline, Embase, and Cumulative Index of Nursing and Allied Health Literature. The eligibility criteria were chronic wound patients with an intervention that involved a comparison of any maggot species with hydrogel dressings. Results: The full text of five studies, involving 580 patients with chronic wounds, was retrieved. Four studies used the *Lucilia sericata* species. The maggot therapy facilitated faster and more effective debridement of non-viable tissue. It enabled faster development of granulation tissue and increased reduction in the wound surface area compared to hydrogel dressings. Maggot therapy had no effect on disinfection or complete healing rate for the wound. Conclusion: Maggot therapy should be considered for faster wound debridement, granulation tissue development, and wound surface area reduction as well as in surgical contraindications. This review can be used as a guide to assist clinicians in identifying patients who may benefit from maggot therapy.

## 1. Introduction

Globally, it is estimated that 1–2% of the population in developing countries experience a chronic wound during their lifetime [1], making it a silent epidemic. The prevalence is increasing with the dramatic increase in the aging population, as wound healing is negatively associated with age [2]. Estimates suggest that 6.6% of the Malaysian populations are over 65 years, and Malaysia is facing the prospect of an aging population as soon as 2030 [3].

In this paper, a chronic wound is one that has failed to proceed through an orderly and timely reparative process to produce anatomic and functional integrity over a period of three months [4]. The typical wound healing process is influenced by extrinsic and intrinsic factors [5], which can be systemic or local [6]. These factors can result in delayed healing and the development of a chronic wound [7]. 

An intervention that continues to attract interest in the treatment of chronic wounds is the use of maggot therapy (MT), also known as maggot debridement therapy or larval therapy [8,9]. Maggot therapy involves the deliberate utilization of live, medical-grade fly larvae for the process of wound healing, disinfection, and the debridement of wounds [10]. Maggots can be applied either in loose (confinement) or bagged (containment) dressings [11], which are non-operator-dependent [12]. 

Although the use of MT is on the rise due to its safety, efficacy, and simplicity, evidence is required before incorporating MT into a patient’s plan of treatment. This is necessary to assist clinicians in determining at which stage of wound healing the treatment should be administered, for what purpose, and when to stop the treatment. Other considerations include the form of treatment to be provided (whether loose or bagged), how the treatment outcomes should be measured and quantified, as well as any possible side effects. This is all crucial information that should be delivered to the patients before initiation of the therapy. As chronic wounds require long-term care, the use of this therapy has the potential to have a significant impact on healthcare systems. This review provides a reliable source of information to policymakers on the use of MT. The aim of the review is to compare the effect of MT with that of hydrogel dressings with patients with chronic wounds. 

## 2. Methods

An electronic literature search was performed using Medline, Embase, and Cumulative Index of Nursing and Allied Health Literature, from inception until October 2019. The search was performed using the mesh terms and text words of “larva”, “maggot therapy”, “chronic wound”, “wound healing”, “biosurgery”, “debridement”, “granulation tissue”, and “disinfection”. We searched for ongoing trials through the World Health Organization International Clinical Trials Registry Platform. 

The criteria for the eligibility of studies were patients with chronic wounds (such as diabetic foot ulcers, pressure injuries, and vascular ulcers) and an intervention involving any species of maggot, which was compared with the use of hydrogel dressings. Combinations of MT with other interventions and studies with maggot-derived substances were excluded. The clinical outcomes were measured by the effects of the MT on the debridement of non-viable tissue, disinfection of bacterial growth, growth of granulation tissue, reduction in wound surface area, complete healing, adverse events, and the duration of the healing process. The search was restricted to the English language.

The review authors scanned the titles and abstracts obtained from the searches and obtained the full-text articles when they appeared to meet the eligibility criteria. The eligibility of the studies was assessed, and reasons for exclusion were documented. Any disagreements between the review authors were resolved by the third author. The Critical Appraisal Skills Programme (CASP) tool was used to appraise the quality of evidence of the included studies [13], and the National Health and Medical Research Council (NHMRC) levels of evidence were used to rank the evidence [14].

We assessed for risk of bias as outlined in the Cochrane Handbook of Systematic Reviews of Interventions [15]. Two review authors independently assessed each trial based on selection bias (sequence generation, allocation sequence concealment), performance bias (blinding of participants and personnel), detection bias (blinding of outcome assessment), attrition bias (incomplete outcome data) and reporting bias (selective outcome reporting). We categorized the risk of bias as low, unclear, or high.

## 3. Results 

**Figure 1:**
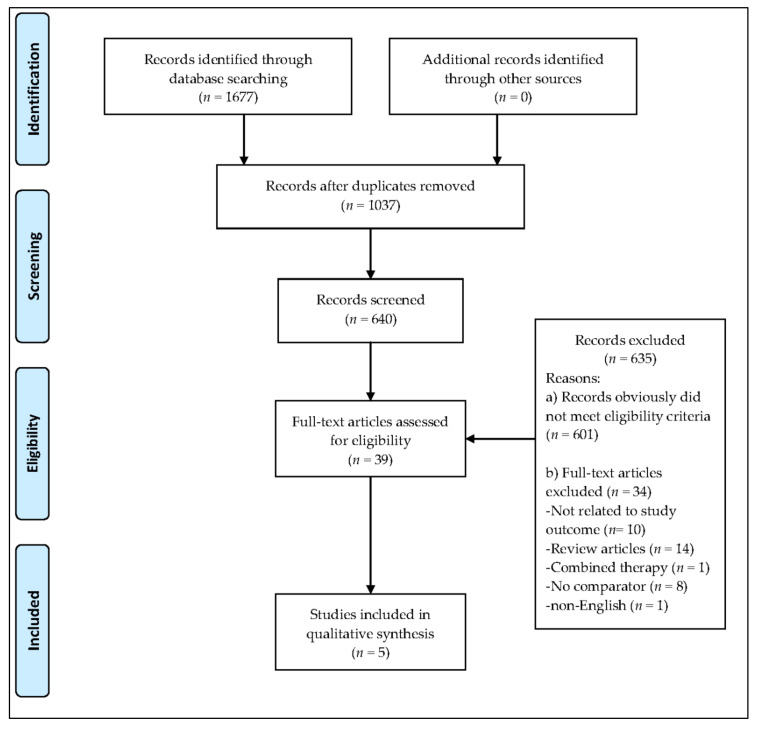
Preferred Reporting Items for Systematic Reviews and Meta-Analyses flow chart.

**Figure 2:**
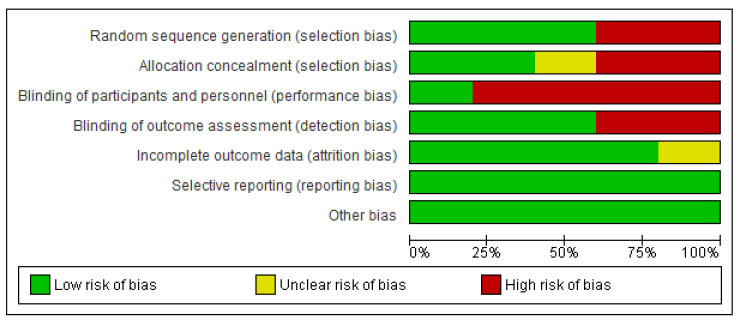
Risk of bias graph.

**Figure 3:**
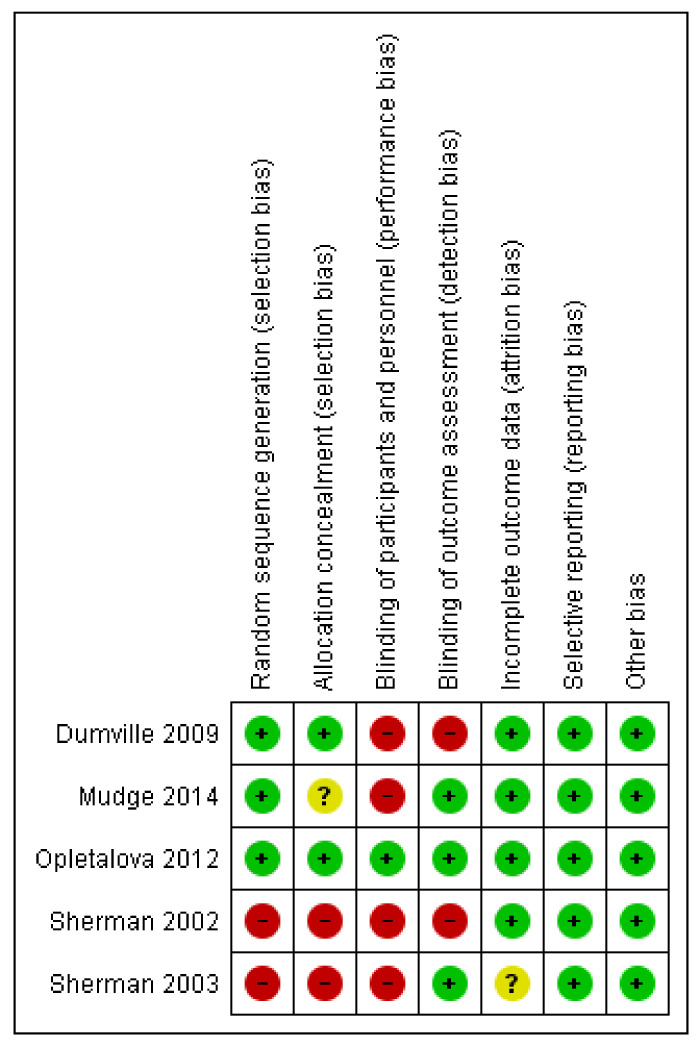
Risk of bias summary. The green color indicates low risk of bias, yellow color indicates unclear risk of bias and red color indicates high risk of bias.

The database search resulted in the identification of 1677 records (Figure 1). In total, 1037 duplicates were removed, and 601 studies were excluded based on the eligibility criteria. Subsequently, 39 full-text articles were identified, of which 34 articles were removed due to reasons that included the article being written in a language other than English, the use of hydrogel dressings not being examined, or combinations of MT with other interventions. No systematic review or meta-analysis relating exclusively to the effectiveness of MT in chronic wound healing and comparing MT with the use of hydrogel dressings was identified. No grey literature or current clinical trials in progress were found. The full texts of the five studies evaluating the effectiveness and safety of MT in the treatment of chronic wounds are summarized in Table 1. The findings of the five studies, based on the nine questions on the CASP checklist, are summarized in Table 2. Figure 2 and Figure 3 represent the risk of bias graph and summary of the included studies, respectively. 

### 3.1. Participants

Five studies were identified for the review with a total of 580 participants [12,16,17,18,19]. Of the five studies, three were randomized controlled trials (RCTs) [12,18,19], one was a retrospective trial [17], and one was a prospective cohort study [16]. Two studies were conducted in the USA [16,17], two in the UK [18,19], and one in France [12]. The number of samples in the studies ranged from 28 [17] to 267 [18]. Two studies [16,17] included patients with more than one wound. 

The studies investigated patients with venous ulcers [12,18,19], mixed leg ulcers [18,19], pressure ulcers [16], and diabetic foot ulcers [17]. The ankle brachial pressure index (ABPI) was assessed, and levels ≥ 0.5 [19], ≥ 0.6 [18] and ≥ 0.8 [12] were included in the respective studies. All studies declared their sources of funding, and one study [19] received funding from a European wound care company, which is also a manufacturer and distributor of MT products, Biomonde Ltd, Bridgend, United Kingdom. However, the authors declared that there was no conflict of interest.

### 3.2. Intervention

The studies followed up with patients for 3 to 12 months [18], 19 weeks [16], 8 weeks [17], 3 to 12 months [18], 35 days [19], and 30 days [12]. Four studies used the *L. sericata* species of larvae [12,16,17,18], and one study [19] did not report the species of larvae used. Different numbers of larvae were applied to the wounds. Two studies [16,17] used loose larvae and applied five to eight larvae to every square centimeter of wound. Dumville et al. [18] used both loose and bagged larvae and referred to a loose larvae calculator to decide on the number of larvae. Two recent studies [12,19] used bagged larvae. Opletalová et al. [12] described that each bag for the MT contained 80 maggots, but Mudge et al. [19] did not mention any number.

The duration for the application of larvae onto the wound differed between the studies. The larvae were left in situ for 48 h [16,17] and three to four days [18,19] prior to the removal of the dressing. Opletalová et al. [12] did not report on the duration of the applications; however, they did mention that MT was performed twice a week, and that larvae could be left for up to four days.

### 3.3. Comparisons

A limited number of studies exclusively compared MT with hydrogel dressings. Two studies [18,19] used Purilon hydrogel dressings for the control group. Opletalová et al. [12] used surgical debridement as the main comparator, and after the debridement, the patients received either hydrogel covered with a hydrocolloid dressing for dry wounds or alginate or fiber-based dressing for oozing wounds. Two studies [16,17] used a variety of dressing materials, based on the condition of the wound. These included Acemann and other hydrogels, growth factors, hydrocolloids, calcium alginates, chemical debriding agents, saline-moistened or “wet-to-dry” dressings, multiple combinations of nonsurgical treatments, topical antimicrobial therapy, and intraoperative or bedside surgical debridement. Sherman [16] reported that only 10% of the wounds studied received hydrogel dressings. 

Apart from the standard application of hydrogel dressings, other concurrent interventions were used. Two studies [18,19] applied compression to their patients after application of MT and hydrogels. Dumville et al. [18] performed compression during Phase II of their study only.

### 3.4. Outcomes 

The summary of clinical outcomes assessing the effects of MT on the debridement of non-viable tissue, disinfection of bacterial growth, growth of granulation tissues, reduction of wound surface area, complete healing, adverse events, and the duration of the healing process is shown in Table 3.

#### 3.4.1. Debridement of Non-Viable Tissue

Overall, the evidence indicates that MT is favorable in the debridement of chronic wounds [12,16,17,18,19]. Sherman [16] reported that MT completely debrided necrotic tissue in less than five weeks, whereas Sherman [17] reported similar findings after four weeks.

Dumville et al. [18] reported that MT reduced the time for the debridement of necrotic and sloughy chronic venous and mixed venous/arterial leg ulcers compared to the hydrogel treatment. Likewise, Opletalová et al. [12] reported that there was statistically significant faster debridement of slough tissue, but only for the first week of MT. A later study by Mudge et al. [19] found a statistically significant difference in the number of completely debrided wounds in the MT group compared to the hydrogel, which indicates that MT leads to faster debridement. 

#### 3.4.2. Disinfection of Bacterial Growth

Disinfection of bacterial growth is evidenced by the elimination of the pathogenic microorganisms, except bacterial spores, on the wound bed [20]. Three [12,18,19] of the five studies reported that there was no statistically significant effect in the disinfection of bacterial growth between the MT and hydrogel dressings. Two studies [16,17] did not measure this outcome.

#### 3.4.3. Growth of Granulation Tissue

Two studies [16,17] suggested that MT hastens the growth of granulation tissue. Sherman [16] reported that ulcers treated with MT attained at least 50% of granulation tissue within three weeks, whereas Sherman [17] found that the amount of healthy granulation tissue was statistically significant after complete debridement was achieved in four weeks. Conversely, Mudge et al. [19] suggested that there was no statistically significant difference in the growth of granulation tissue. While this study is of a superior level and quality, it had a relatively small sample size for the final analysis (*n* = 42) due to high withdrawal. Two studies [12,18] did not investigate differences in the growth of granulation tissue.

#### 3.4.4. Reduction of Wound Surface Area

Three studies [12,16,17] concluded that MT assisted in the faster reduction of wound surface area. Sherman [16] found that wounds in the group that had MT had a total wound surface area decrease of 1.2 cm^2^/week, whereas Sherman [17] reported that the wound surface area decreased by 0.9 cm^2^/week during MT. A later study found that the reduction in the wound surface area was significantly better in the MT compared to conventional therapy group at day 15, but the healing rates were not significant at either day eight or day 30 [12]. Two studies [18,19] did not measure the reduction in wound surface area. 

#### 3.4.5. Complete Healing

Three studies [16,17,18] found that wounds treated with MT healed completely faster than those treated with hydrogel dressings; however, the difference was not statistically significant in comparison to the control group. Two studies [12,19] did not investigate the effect of MT on the complete healing of the wounds. 

#### 3.4.6. Adverse Events

None of the studies reported significant serious adverse effects during the MT. Three studies used the visual analog scale for pain, two of which [18,19] reported pain as a significant side effect of the MT, while one study did not [12]. Dumville et al. [18] described that patients had experienced significantly more pain in the previous 24 h at the removal of the first debridement treatment, while Mudge et al. [19] found more ulcer-related pain or discomfort in the MT group. In the other two studies [16,17], two patients complained of mild pain during MT, but the same patients had similar pain during the conventional treatment. No tool was specified for the assessment of pain in two studies [15,16].

#### 3.4.7. Duration of Healing

Three studies [16,17,18] reported on the duration of healing with the MT. However, none of the findings were significant. Sherman [16] reported that wounds treated with maggots were nearly twice as likely to heal, and that most healed within 12 weeks. Likewise, Sherman [17] found that the average time until wound closure in the MT group was 15 weeks. However, Dumville et al. [18] reported that the median healing time was 236 days (approximately 34 weeks) for the MT group. 

## 4. Discussion 

### 4.1. Summary of Main Results

This study evaluated the available literature in order to establish the effectiveness of MT as compared to hydrogel dressings. As a result, a small number of studies have yielded favorable debriding outcomes in the use of MT. However, more extensive, more suitably powered comparative effectiveness studies are yet to be forthcoming to determine exact clinical effectiveness of MT. Chronic wounds treated with MT can attain more granulation tissue and a faster reduction in the wound surface area. However, no effect was found on the disinfection of bacterial growth or on the duration of complete healing.

### 4.2. Overall Completeness and Applicability of Evidence

The follow-up period varied from 30 days [12] to 12 months [18]. A longer follow-up period allows clinical observation of the entire mechanism of the healing process, that is, debridement, disinfection, and growth of granulation tissue until the wound is completely healed. A 45-week cohort study involving 111 diabetic patients found that the wounds of more than half the patients (*n* = 64) were healed by the end of the study. The median time to healing was 14 weeks [21]. Based on these findings, there were no obvious differences between the approaches that used loose or bagged larvae. A common adverse effect reported by the patients was pain [22]. Almost 30% of patients reported significant pain, especially after 24 h of the MT, which seemed to be related to the increased size of the larvae [23].

Most of the studies included used the *L. sericata* species of larvae, and none reported using *L. cuprina* species, which is the only species used for MT in Malaysia. A study in South Africa found that *L. sericata* and *L. cuprina* differed in their ability to clean unhealthy tissue around wounds [24]. However, one case-control study comparing *L. cuprina* larvae and conventional debridement alone in diabetic foot wounds found that *L. cuprina* is as effective as conventional debridement [25]. Therefore, the applicability of various larvae species regarding their effectiveness and safety for MT should be considered.

### 4.3. Quality of the Evidence

The studies included in this review were generally primary studies at level II [12,18,19] and level III-2 [16,17], which are considered to be Grade A (excellent) and Grade B (good), respectively, as based on the level of evidence provided for the development of clinical guidelines [14]. Grade A provides evidence that can be applied directly in the national healthcare context, whereas Grade B evidence is applicable in the national healthcare context with a few caveats. The review is limited by the small number of included studies. The majority of the studies have a high risk of performance bias due to lack of blinding of participants and personnel.

### 4.4. Agreements and Disagreements with Other Studies or Reviews

There are several reviews on the wound healing of chronic ulcers. One review included three studies on the use of larvae. These studies were of low methodological quality, and the lack of baseline data on the type of wound made it impossible to assume an obvious benefit from the larval healing therapy [26]. The ten studies included in a review of studies concerning debridement for venous leg ulcers represented 715 participants. This is considered a small number of participants when one considers that venous ulcers affect up to 1% of the population, i.e., approximately 600,000 people annually in the USA alone. There is limited evidence to suggest that actively debriding a venous leg ulcer has a clinically significant impact on its healing. A comparison of larval vs. hydrogel debridement shows statistically significant results for the number of wounds debrided [5]. 

A review of studies on chronically infected wounds and ulcers found that MT increased the healing rate and reduced the healing time compared to conventional therapies. However, the findings were limited due to the small number of samples and the various therapies used in the control groups, which may influence the estimation of MT effectiveness. MT is also used as a salvage tool when almost all else has failed to lead to unbiased healing outcomes [27]. There was a 20% greater chance of wound healing for chronic ulcers using MT compared with conventional therapies [21]. A systematic review that included three randomized clinical trials and five non-randomized studies found that MT was significantly more effective as a debriding agent than hydrogel or a mixture of conventional therapy modalities, namely, hydrocolloid, hydrogel, and saline moistened gauze [28].

### 4.5. Potential Biases in the Review Process

Sample size calculation is essential in any clinical study in order to determine whether the sample is large enough to detect the treatment effect with high power [29]. It helps the reader to identify the minimum difference or the effect and importance of the intervention [30]. Researchers may wrongly interpret their study results as significant, but there is a potential type I error when the sample size is inadequate [31]. Three of the later studies [12,18,19] in this review performed power sample calculations. With proper sample size calculations in well-designed RCTs, as covered in this review, the overall risk of type I error is reduced. Only studies on MT with a comparator (hydrogel) were included for analysis. This limited the studies included to only certain RCTs and case-control trials. 

The different comparators used by the different studies in this review make it difficult to draw a firm conclusion regarding the findings. Concurrent interventions, such as compression therapy, are considered the gold standard in ulcer healing [32]. This review is limited to publications in the English language; however, there is no evidence of a systematic bias in the use of language restrictions in systematic review-based meta-analyses in conventional medicine [33].

There are several clinical implications. A standardized clinical practice guideline for the application of MT in terms of the number of larvae, duration, and frequency of dressing is indicated. It should be considered a reasonable alternative in patients with chronic wounds and a primary option for those who are not fit for surgical operation or are in low resource settings. There were no serious adverse effects from the MT, indicating it is a safe intervention for wound healing, thus alleviating concerns for the safety of patients. 

Future studies on the effectiveness of MT should be carefully designed together with concurrent interventions, especially for chronic conditions in routine care settings. Improved research methodologies with larger sample sizes and longer follow-up periods for study participants should be mandatory in experimental designs to determine the average duration for complete healing, which could be of great advantage for future research. Other larvae species, using both the confinement and the containment larvae approaches, should be tested with diabetic foot ulcers and pressure ulcers. Future studies should also focus on treating peripheral arterial disease in patients with ABPI < 0.5, which is most often associated with pain, poor vascularization, and concurrent infection. A survey on the implementation of MT in clinical practice in terms of patient satisfaction and patient experience of care is suggested. 

## 5. Conclusions 

The results of the review need to be interpreted with caution. This is because there is a limited number of level II studies that have shown positive results in the use of MT for debriding devitalized tissue compared to standard care, with results differing across wounds of different etiology. 

## Figures and Tables

**Table 1:** Summary of included studies.

Year	Type of Wound	Number of Participants	Intervention and Control	Outcome	Follow-Up	Level of Evidence
Sherman [16]	Pressure ulcers	MT (loose) = 43 Control = 49	MT vs. “conventional Therapy”	Debridement Granulation Tissue Complete healing Surface area Adverse effects	17–19 weeks	Level III-2
Sherman [17]	Diabetic foot and leg ulcers	MT (loose) = 14 Control = 14	MT vs. “conventional therapy”	Debridement Granulation Tissue Complete healing Surface area Adverse effects	8 weeks	Level III-2
Dumville et al. [18]	Venous or mixed ulcers (ABPI *≥* 0.6)	MT 180 (loose = 94, bagged = 86) Control = 87	MT vs. hydrogel	Complete debridement Bacterial growth Complete healing Adverse events Duration of healing	6–12 months	Level II
Opletalová et al. [12]	Venous leg ulcers (ABPI *≥* 0.8)	MT (bagged) = 51 Control = 54	MT vs. “conventional therapy”	Debridement Wound surface area Bacterial growth Adverse events	30 days	Level II
Mudge et al. [19]	Venous or mixed leg ulcers (ABPI *≥* 0.5)	MDT (bagged) = 46 Control = 42	MT vs. hydrogel	Debridement Bacterial growth Granulation tissue Adverse events	28–35 days	Level II

MT, maggot therapy; ABPI, Ankle Brachial Pressure Index; Level II, evidence obtained from at least one properly-designed randomised controlled trial; Level III-2, evidence obtained from comparative studies with historical control, two or more single arm studies, or interrupted time series without a parallel control group.

**Table 2:** Summary of Critical Appraisal Skills Programme checklist for the studies included.

CASP Checklist	Sherman [16]	Sherman [17]	Dumville et al. [18]	Opletalová et al. [12]	Mudge et al. [19]
Are the results valid?					
Focused issue ^1^	Yes	Yes	Yes	Yes	Yes
Randomization ^2^	No	No	Yes	Yes	Yes
Eligibility ^3^	Yes	Yes	Yes	Yes	Yes
Blinding ^4^	No	No	No	Yes	Cannot tell
Baseline homogeneity ^5^	Cannot tell	Cannot tell	No	Yes	Yes
Equal treatment ^6^	Cannot tell	Cannot tell	Yes	No	Yes
Will the results help locally?					
Application of results ^7^	Yes	Yes	Yes	Yes	Yes
Important outcomes ^8^	Yes	Yes	Yes	Yes	Yes
Benefits ^9^	Yes	Yes	Yes	Yes	Yes

CASP, Critical Appraisal Skills Programme. ^1^ Did the trial address a clearly focused issue? ^2^ Was the assignment of patients to treatments randomized? ^3^ Were all of the patients who entered the trial properly accounted for at its conclusion? ^4^ Were patients, health workers and study personnel ‘blind’ to treatment? ^5^ Were the groups similar at the start of the trial? ^6^ Aside from the experimental intervention, were the groups treated equally? ^7^ Can the results be applied to the local population, or in your context? ^8^ Were all clinically important outcomes considered? ^9^ Are the benefits worth the harms and costs?

**Table 3:** Summary of outcomes.

Study Objectives	Sherman [16]	Sherman [17]	Dumville et al. [18]	Opletalová et al. [12]	Mudge et al. [19]
Debridement of the non-viable tissue	Significant (*p* = 0.021) (Faster)	Significant (*p* = 0.001) (Faster)	Significant (*p* < 0.001) (Faster)	Significant (*p* = 0.040) (Faster)—For the first one week	Significant (*p* = 0.001) (Faster)
Disinfection of bacterial growth	Not studied	Not studied	Not significant	Not significant	Not significant
Growth of granulation tissue	Significant (*p* < 0.001) (Faster)	Significant (*p* = 0.016) (Faster)	Not studied	Not studied	Not significant
Reduction on wound surface area	Significant (*p* = 0.001) (Faster)	Significant (*p* = 0.018) (Faster)	Not studied	Significant (*p* = 0.020) (Faster)	Not studied
Complete healing	Not significant	Not significant	Not significant	Not studied	Not studied
Adverse events	Not significant	Not significant	MT significantly more painful (*p* < 0.001)	Not significant	MT significantly more painful (*p* < 0.001)
Duration of healing	Within 12 weeks	Within 15 weeks	Within 236 days	Not studied	Not studied
